# Construction of PARI public health education programs for Chinese undergraduates: a Delphi study

**DOI:** 10.3389/fpubh.2024.1390011

**Published:** 2024-06-17

**Authors:** Yuzhe Kong, Haitao Xu, Chuyan Li, Yang Yang, Xiaoyi Zhu, Yu Zuo

**Affiliations:** ^1^Xiangya School of Medicine, Central South University, Changsha, Hunan, China; ^2^Xiangya School of Public Health, Central South University, Changsha, Hunan, China; ^3^Third Xiangya Hospital, Central South University, Changsha, China

**Keywords:** PARI, construction, public health education, undergraduates, Delphi

## Abstract

**Objectives:**

The objective of this study is to develop a consensus among experts on a comprehensive and scientifically sound physical activity-related injuries (PARI) public health education program specifically tailored for undergraduates.

**Methods:**

This study designed three rounds of expert consultation by using a Delphi method. A panel of 30 experts from the fields of public health education, sports medicine, anesthesia pain, emergency medicine, and emergency nursing participated in the study.

**Results:**

This study successfully established a consensus among experts on the goals, content, teaching methods, and time allocation for the PARI Public Health Education Program for undergraduates. The program encompasses 10 objectives divided into 2 main categories: professional knowledge and skill goals. In terms of content, it includes 5 primary indicators, 22 secondary indicators, and 56 detailed tertiary indicators. Six teaching methods were identified as suitable. Additionally, a typical 60-min educational session was segmented into eight parts, with a proposed time arrangement for each, ensuring comprehensive coverage of all topics.

**Conclusion:**

The consensus achieved in this study on the PARI Public Health Education Program for undergraduates lays a crucial foundation for the advancement of health literacy and proactive health management within this demographic. We presented a comprehensive framework for PARI public health education, integrating diverse learning methods and content areas. This systematic approach not only enriched the resources available for undergraduate health education, especially of PARI but also had the potential to significantly impact the implementation and effectiveness of health promotion strategies.

## 1 Introduction

In recent years, the focus on health and fitness has surged significantly, keeping pace with the growing realization of the benefits of sports and physical activities (1). However, this surge in participation has led to a notable rise in the number of physical activity-related injuries (PARI). As of 2022, 67.5% of the Chinese population aged 7 and older engaged in physical exercises at least once a week, marking an 18.5% increase from 2014 (2). The rate of PARI in China stands at approximately 10–20% and continues to climb. The growing popularity of marathons and other running events has drawn more people into running, but this trend has also led to an escalation in PARI (3, 4).

The PARI refer to sudden bodily injuries that occur during sports or exercise, such as bruises, strains, sprains, and fractures. These injuries often result from accidental or sudden physical impacts, improper exercise techniques, misuse of equipment, or unsafe sports environments (5, 6). Regular cumulative sports injuries, in contrast, develop gradually due to repetitive stress or overuse of specific body parts, typically involving overstrained muscles, tendons, and joints. An example is knee arthritis from long-term running. Unlike cumulative injuries, PARI usually require timely and accurate medical assessment and intervention to prevent further damage and aid recovery. If not addressed promptly, PARI can be life-threatening or leave long-term physical complications for the patient (7–10).

Internationally, the preventive measures for PARI are well-developed, resulting in a high level of awareness and knowledge about PARI prevention among active populations (11). Countries like the United States, United Kingdom, Canada, and Australia have published relevant documents educating the public on methods to prevent PARI. For example, researchers have evaluated the effects of preventive interventions on sports injuries in children and adolescents, concluding that such measures are very effective (12). Consistent recommendations for reducing injury risk include having properly trained staff and sessions that include a warm-up, cool-down and skills progression (through the session) appropriate to the level of the participants to teach the public about exercise methods (13).

In China, the general public’s understanding of PARI is not high. As university students are among the more knowledgeable groups in the public, they should have a better understanding of PARI. However, the articles cited point out that the overall risk of injury for sampled Chinese university students are 0.34 and 0.27 injuries/students/year respectively, indicating a high risk associated with PARI (14, 15). Currently, China has developed a relatively comprehensive pre-hospital emergency care system for PARI, with doctors and emergency personnel possessing a high level of knowledge in PARI prevention and treatment (16–18). However, to date, there has been no research focusing on public awareness or education regarding the prevention and treatment of PARI, leaving the public’s understanding in this area unknown. For the Chinese public, PARI poses a significant threat to their physical health, especially for university students who frequently engage in sports activities, thus facing substantial risks. Therefore, we target university students first in launching a science popularization program on PARI.

The Delphi technique is a structured approach that employs a series of questionnaires to collect information (19). This process continues through multiple rounds until a group consensus is achieved. One of the key reasons for the Delphi technique’s popularity is its ability to include a large number of participants from diverse locations and areas of expertise anonymously (20). This prevents any single expert or small group of experts from dominating the consensus process.

Therefore, this study plans to use the Delphi method to construct and implement an public health educational program for the prevention and treatment of PARI, aiming to ensure the life safety and physical health of the public when affected by PARI.

Compared to the numerous training programs targeting doctors and emergency personnel for emergency care skills, this research is the first in China to focus on educating the public about PARI prevention and treatment. Innovatively applying the Delphi method in developing public educational programs, this study aims to create a more scientific and systematic approach, thereby ensuring the life safety and physical health of the Chinese population following PARI incidents.

## 2 Materials and methods

In this study, the Delphi method is the primary approach utilized. Commonly employed in fields such as education and health, this method leverages expert knowledge to develop and refine new research topics. The Delphi technique is particularly apt for this study’s aims.

### 2.1 Study design

Firstly, this study included experts from the fields of public health education, sports medicine, anesthesia pain, emergency medicine, and emergency nursing sourced from the CNKI database. Experts who met the inclusion criteria used to conduct in-depth research and publish articles in their respective fields. Subsequently, we draft the first round of expert questionnaires and sent them to the experts. Based on the results of the first round of responses from the expert panel, we revised the objectives and contents of the PARI public health education program and modified it to form the second round of expert questionnaires, which were then sent out. After that, we conducted a third round of inquiries and finalized the PARI public health education program.

### 2.2 Ethic approval

Ethical approval regarding human subject research was obtained from the Ethics Committee on Third Xiangya Hospital of Central South University (approval number: Fast24084). Informed consent was obtained from each participant offline by placing a question about their agreement to participate in the study at the beginning of the survey. Participants were assured of the confidentiality and anonymity of this study and their rights to exit at any time. We declare that the data were collected for academic use only.

### 2.3 Expert selection

Experts in the fields of public health education, sports medicine, anesthesia pain, emergency medicine, and emergency nursing from the CNKI database were included in this study. CNKI is a major academic online library launched in 1996 by Tsinghua University and Tsinghua Tongfang Company. It’s the largest repository of Chinese academic resources, offering access to journals, dissertations, conference proceedings, and more. Primarily used in China, CNKI supports research, education, and learning by providing extensive scholarly materials predominantly in Chinese.

Inclusion criteria were as follows: (1) master’s degree or higher; (2) intermediate or higher title; (3) more than 5 years of work experience.

Exclusion criteria were as follows: (1) experts who could not participate in this study due to personal reasons; (2) experts whose contact information was unknown; (3) experts who had no practical experience in geriatric work.

The experts who met the inclusion criteria had conducted in-depth research and published articles in the relevant fields. Therefore, email addresses and some phone numbers could be obtained from the database. The experts were then invited to participate in the survey by e-mail, telephone or SMS, and they were given a detailed description of the content, purpose and significance of the study.

A total of 30 experts agreed to participate in this Delphi study and a follow-up questionnaire was sent to them and then sent back by e-mail.

### 2.4 Questionnaire design

The questionnaire for the first round for experts consists of five parts: (1) experts’ personal socio-demographic information such as unit and department, sex, age, years of working in first aid science, professional field and so on; (2) self-assessment of the degree of understanding of the consultation; (3) expert views on the degree of impact of the main basis (theoretical analysis, practical analysis, peer understanding and intuition) for judging the content of the consultation; (4) expert evaluation form for the PARI public health education program’s goals; (5) expert evaluation form for the PARI public health education program’s contents, where the fourth and fifth parts are the main body of assessment in the expert questionnaire.

By retrieving related popular science book, we decided to at least include both specialized knowledge popularization and technical ability popularization to form the science popularization program for PARI prevention and management. In this study, through literature reading and group discussion, we proposed the contents of the first draft of the scientific program for the prevention and management of acute sports injuries based on the “Interpretation of the National Fitness Guidelines” and the “Expert Consensus on Exercise Prescribing (2023),” in which there were 15 first-level, 24 s-level, and 67 third-level indexes and the importance of each part was assessed by experts with scores.

A 5-point Likert scale method was employed to measure importance from 5 points to 1 point as “very important”–“very unimportant,” in descending order of importance. Considering the limited duration of the public health education program and the effectiveness of it, the inclusion of items needed to take time and teaching methods factors into account. Therefore, the questionnaire for the second round added items related to teaching time and teaching methods.

Additionally, an item regarding approval rate was included. There were no changes in the third round.

### 2.5 Questionnaire procedures

In this study, three rounds of questionnaires were distributed to the panel of experts, who met face-to-face with the researcher offline. There was no communication between the experts during this process. The first round of the survey summarized two primary and several secondary objectives, as well as primary, secondary and tertiary structures. In addition to scoring and evaluating the importance of each element, the experts were able to provide their own professional opinions and suggestions for modifying, deleting, and adding other relevant elements to enrich the PARI Preventive and Management Sciences curriculum.

Based on the results of the first round of questioning by the expert group, the objectives and contents of the PARI public health education program were revised. Meanwhile, in order to make the study more practical, the PARI Public Health Education Program Teaching Methods and Timing Scale was added to the second round of questionnaires. Feasibility was also measured using a 5-point Likert scale method, with a score of 5 to 1 being “feasible” – “infeasible,” and “feasible” – “infeasible” in descending order.

The four sections of the questionnaire were deleted and supplemented based on the comments from the second round of the survey and the objectives of the study. Eventually, an expert assessment form for the third round of the survey was developed, and experts’ comments and feedback were collected and analyzed.

Particularly worth mentioning is the addition of an overall evaluation scale for the public health education program at the end of each questionnaire, so that experts could evaluate the program’s scientific and operability as well as the clarity of the subject matter and help us improve the study.

### 2.6 Data analysis

The returned data were analyzed using R 4.3.2. Experts’ concern for this study was reflected in the positive coefficient of the expert panel, i.e., the rate of return of a questionnaire (21). It is generally believed that the positive coefficient above 85% indicates good feedback of the survey from the expert panel.

The representativeness of the experts in this research were presented as expert authority coefficient (21). Generally, an authority coefficient over 0.70 indicates that the expert opinions are reliable and the experts are authoritative.

The inclusion criteria for items in this study were as follows: (1) the average importance score of each item evaluated by the expert panel >4.00; (2) the coefficient of variation <0.20; (3) the approval rate >80% (if applicable) (22).

The exclusion criteria for items in this study were as follows: (1) the average importance score of each item evaluated by the expert panel is less than or equal to 4; (2) The coefficient of variation is greater than or equal to 20%; (3) the approval rate is less than or equal to 80% (if applicable); (4) the item is suggested for deletion by 6 or more experts; (5) the item is suggested for deletion by 6 or fewer experts, but its average score in the next round is greater than 0; (6) when a second-level item is deleted, and if its subordinate third-level items are suggested for deletion by 6 or more experts in the next round, then those third-level items are deleted; otherwise, they are moved under another second-level item; (7) when a first-level item is deleted, if all third-level items under a second-level item are deleted, then the second-level item is deleted.

Similarly, if all second-level items under a first-level item are deleted, then the first-level item is deleted. Furthermore, based on some written opinions of the experts with literature retrieval, the proposed contents were discussed among the research team and improved by means of deletion, modification, supplementation, and merger.

## 3 Results

### 3.1 Expert sociodemographic information

This study initially involved a diverse panel of 30 experts from five distinct fields: public health education, sports medicine, anesthesia pain, emergency medicine, and emergency nursing. The experts’ professional experience ranges from 5 to 30 years, and 70% have over two decades of experience in their respective fields. All experts have prior experience in conducting public health education activities. The sociodemographic details of these experts are documented in Table 1.

**Table 1:** The socio-demographic information of the experts.

Groups	Classification	Number of first-round	Percentage	Number of second-round	Percentage	Number of third-round	Percentage
Gender	Male	19	0.63	19	0.63	19	0.63
	Female	11	0.37	11	0.37	11	0.37
Age	30 ~ 39	12	0.40	12	0.40	12	0.40
	40 ~ 49	18	0.60	18	0.60	18	0.60
Years of working	5 ~ 9 years	2	0.07	2	0.07	2	0.07
	10 ~ 19 years	19	0.63	19	0.63	19	0.63
	20 ~ 29 years	9	0.30	9	0.30	9	0.30
Highest education	Master	12	0.40	12	0.40	12	0.40
	Doctor	18	0.60	18	0.60	18	0.60
Professional positions	Intermediate certificate	10	0.33	10	0.33	10	0.33
	Senior position	20	0.67	20	0.67	20	0.67
Supervisor	Doctor supervisor	8	0.27	8	0.27	8	0.27
	Master supervisor	13	0.43	13	0.43	13	0.43
	No	9	0.30	9	0.30	9	0.30
Specialist areas	Popular Science (Internal Medicine, Chronic Disease)	7	0.23	7	0.23	7	0.23
	Popular Science (Surgery)	2	0.07	2	0.07	2	0.07
	Popular Science (Critical Care Direction)	5	0.17	5	0.17	5	0.17
	Sports Medicine	4	0.13	4	0.13	4	0.13
	Anesthesia Pain	1	0.03	1	0.03	1	0.03
	Emergency Medicine	8	0.27	8	0.27	8	0.27
	Nursing (Emergency Care Direction)	3	0.1	3	0.10	3	0.10

The present study involved three rounds of questionnaires to the expert panel. In three rounds of Delphi, 30 experts returned questionnaires in time. In general, the response rate of the experts is 100%, and the authority coefficient is 0.9159 (>0.80), indicating the authority of the experts and the credibility of the Delphi results. Experts were interested in the study through their comments. The main purpose of each round of questionnaires, the number of questionnaire items to be evaluated by experts, item revisions and expert opinions were presented in Table 2.

**Table 2:** The process of items revision.

Round	Response rate	Authority coefficient	Purpose	Total number of items
The first round	100.00	0.9159	Exploration	106
The second round	100.00		Clarification	118
The third round	100.00		Confirmation	107

Round	Number of modified items	Number of deleted items	Number of added items	Number of experts’ suggestions
The first round	4	8	20	64
The second round	1	9	0	70
The third round	0	0	0	3

### 3.2 Evaluation of goals

In the first Delphi round, the panel evaluated the PARI Public Health Education Program’s objectives. Items under ‘Knowledge goals’ and ‘Skill goals’ were assessed. High mean scores close to 5.00 with minimal standard deviation (SD) indicated a strong consensus for items (see Supplementary Table S1). The second round showed a convergence of opinions, with all items achieving a mean score of 5.00, except for ‘Understanding the importance of fitness testing’ and ‘Conducting fitness tests’ which retained a mean of 4.97 but had reached 100% agreement rate (AR) (see Supplementary Table S2). By the third round all items sustained a perfect mean score of 5.00 and the 100% AR (see Supplementary Table S3).

**Figure 1:**
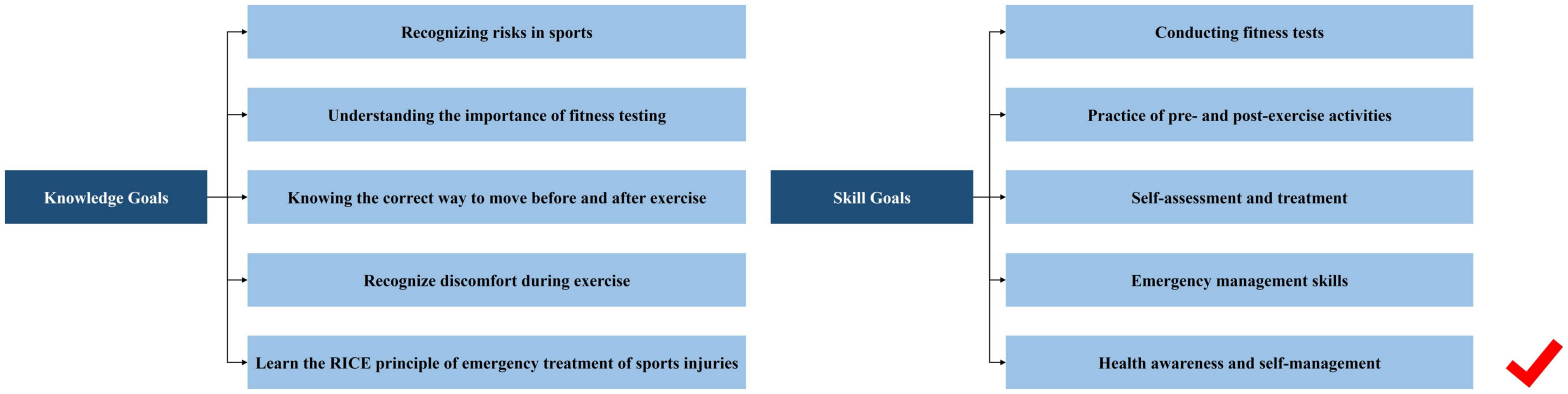
Screening of entries in Delphi’s three rounds of Goals.

Screening of entries in Delphi’s three rounds is shown in Figure 1.

### 3.3 Evaluation of contents

During the initial Delphi round for the PARI Public Health Education Program Contents, varied items across multiple health and fitness domains were scrutinized. While ‘Risk Indicators in Sports’ and ‘Physical Activity Levels’ received moderate scores with means of 4.73 and 4.37 respectively, certain third-level items like ‘Definitions and Importance’ and ‘Recommended Criteria’ under ‘Physical Activity Levels’ were marked for modification due to their lower means and higher coefficients of variation (CV). Conversely, some items like ‘Recognizing Signs and Symptoms’ related to ‘kidney disease’ scored significantly lower, leading to their proposed deletion (see Supplementary Table S4).

Moving into the second Delphi round, there was a noticeable improvement in consensus as indicated by the higher means, lower SDs, and CVs, and increased agreement rates (AR) across most items (see Supplementary Table S5).

In the final round, the expert panel reached full consensus on many key items, signifying a strong expert consensus on the importance and definitions of these concepts within the PARI program (see Supplementary Table S6).

**Figure 2:**
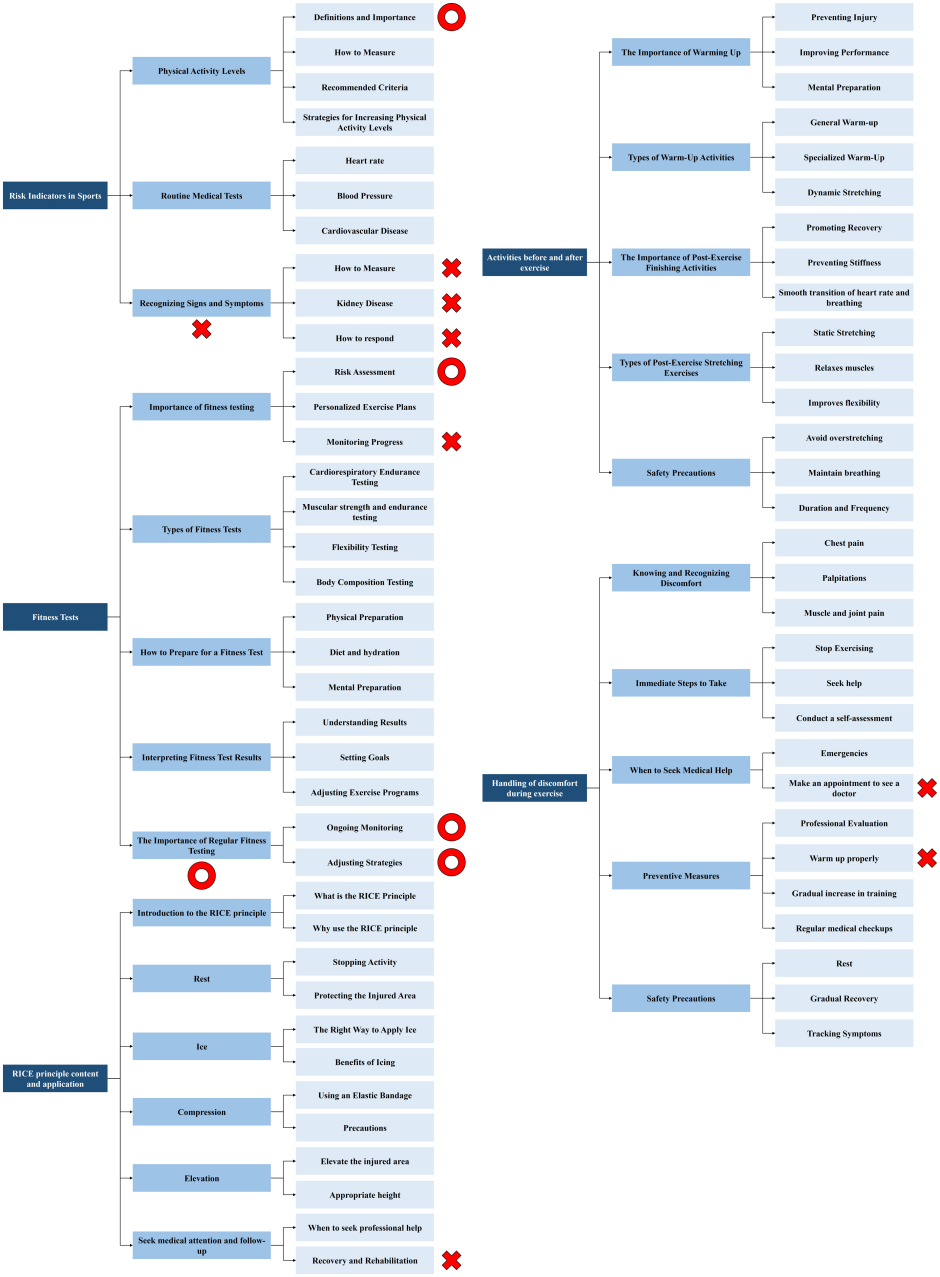
Screening of entries in Delphi’s first rounds of Contents.

**Figure 3:**
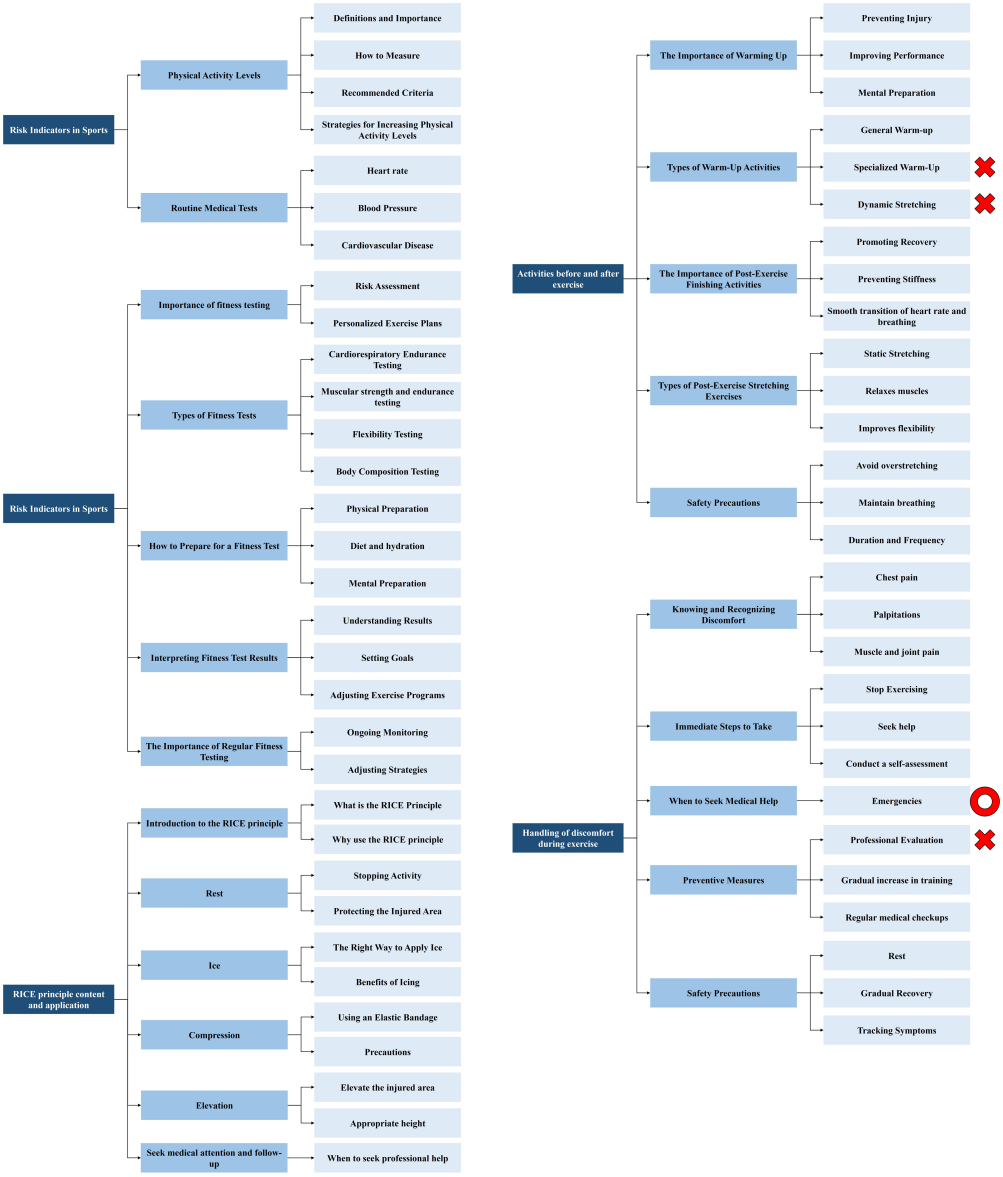
Screening of entries in Delphi’s second rounds of Contents.

**Figure 4:**
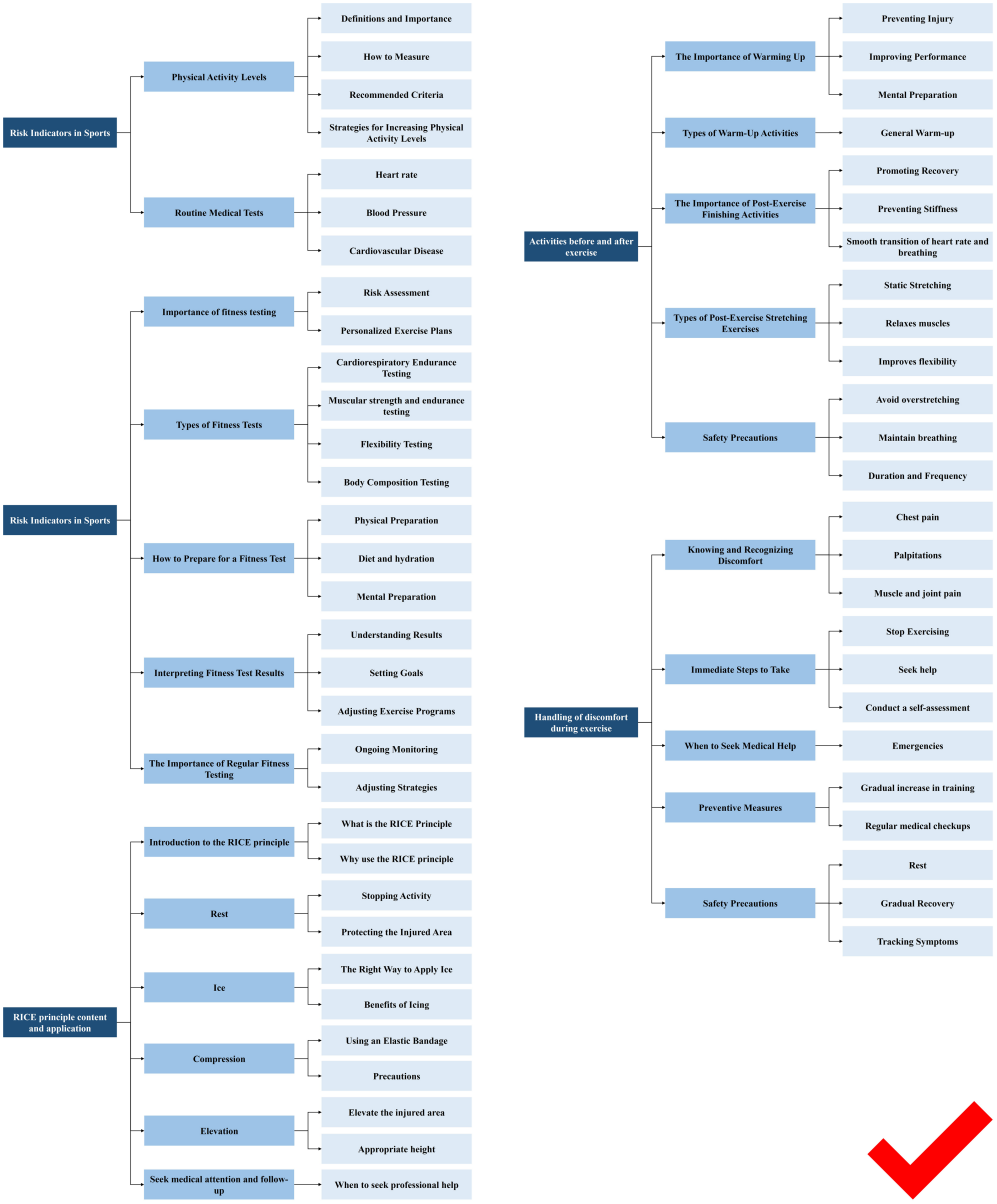
Screening of entries in Delphi’s third rounds of Contents.

Screening of entries in Delphi’s three rounds is shown in Figures 2–4.

### 3.4 Evaluation of teaching methods

From the second round of the Delphi process, various pedagogical strategies for the PARI Public Health Education Program were additionally evaluated. Techniques such as ‘Role Play’ and ‘Interactive Questionnaire’ showed a divergence in expert opinions, as reflected by higher standard deviations and the decision to delete certain methods based on recommendations. Conversely, ‘Experiential Learning’ was unanimously endorsed with a perfect mean score, indicating a strong consensus on its effectiveness (see Supplementary Table S7).

By the third round, after deleting six items, the consensus among the experts had strengthened, with ‘Sports Injury Prevention Workshop,’ ‘Video Analysis and Discussion,’ and ‘Online courses and resources’ receiving near-perfect mean scores and full agreement. The inclusion of ‘Infographics and Visual Aids,’ and ‘Reflections and journal entries’ also demonstrated high mean scores (see Supplementary Table S8).

**Figure 5:**
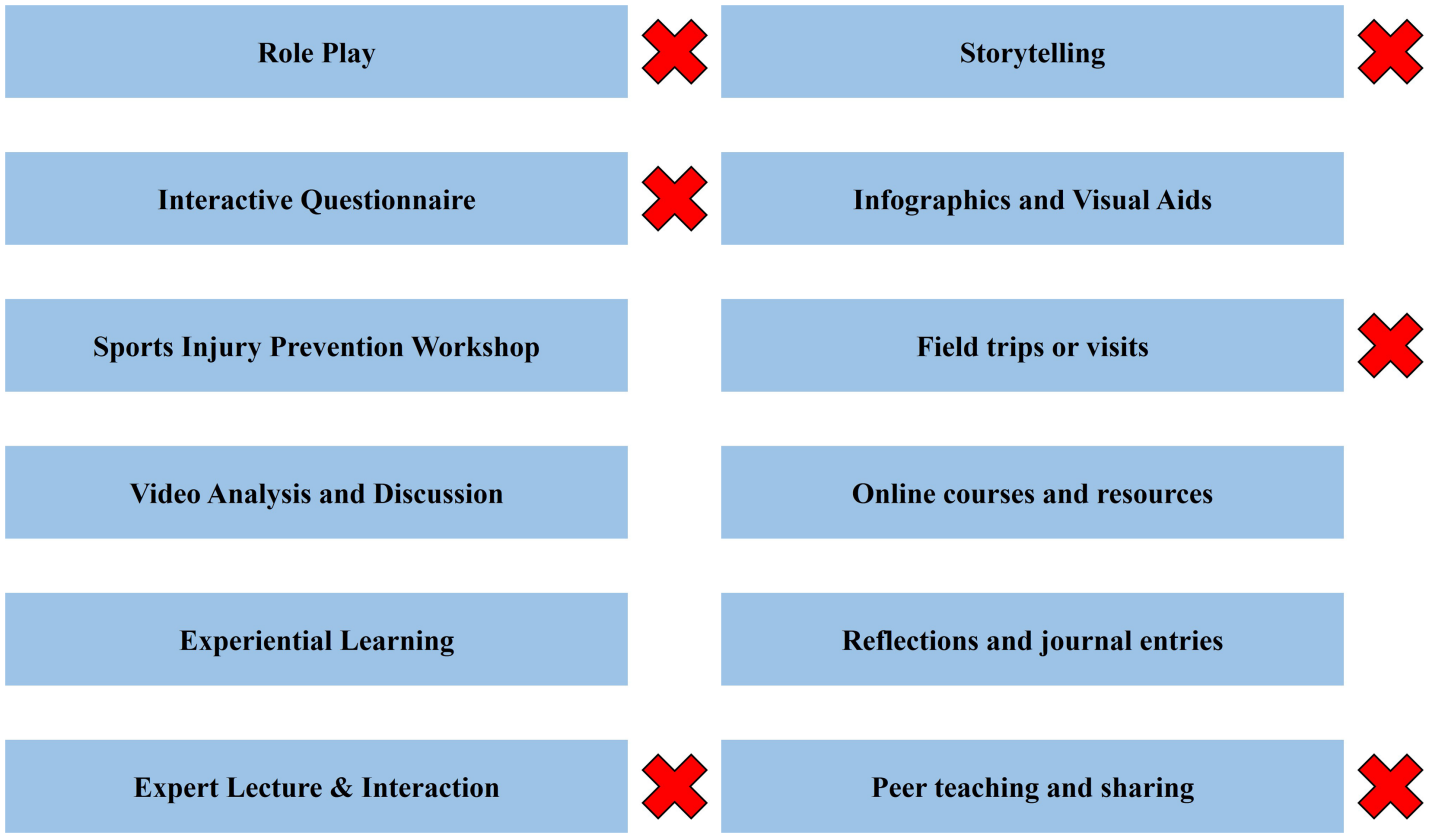
Screening of entries in Delphi’s second rounds of Teaching Methods.

**Figure 6:**
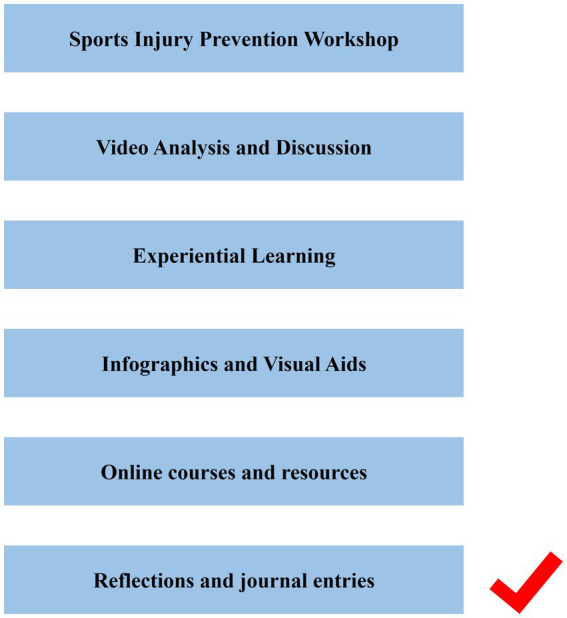
Screening of entries in Delphi’s third rounds of Teaching Methods.

Screening of entries in Delphi’s two rounds is shown in Figures 5, 6.

### 3.5 Evaluation of time arrangement

In the second round of the Delphi process, experts were engaged to estimate the time needed for various components of the PARI Public Health Education Program. The results yielded mean times with standard deviations, allowing for a nuanced understanding of the expected duration for each activity (see Table 3).

**Table 3:** Time arrangement of PARI public health education program after expert consultation (second-round).

Item	Mean	*SD*	CV	Time arrangement	Notes
Activity opening and introduction	4.87	0.35	7.19	9.80 ± 1.85	
Risk of cardiovascular events during exercise and its indicators	4.87	0.35	7.19	19.97 ± 1.87	
Fitness testing and risk reduction	4.90	0.31	6.33	14.63 ± 1.60	
Preparation and recovery activities before and after exercise	4.87	0.35	7.19	15.03 ± 1.74	
Symptoms and emergency treatment during exercise	5.00	0.00	0.00	19.90 ± 1.66	
RICE principle	5.00	0.00	0.00	15.17 ± 2.05	
Interactive Q&A and summary	4.97	0.18	3.62	19.97 ± 1.96	
End of the program and thank you	4.90	0.40	8.16	5.53 ± 1.65	

By the third round, the experts reached a full consensus (100% agreement rate) on the importance of each program component. The time arrangements made in the previous round were not just approved, but strongly endorsed (see Table 4).

**Table 4:** Time arrangement of PARI public health education program after expert consultation (third-round).

Item	Mean	*SD*	CV	AR	Notes
Activity opening and introduction	5.00	0.00	0.00	100.00	
Risk of cardiovascular events during exercise and its indicators	5.00	0.00	0.00	100.00	
Fitness testing and risk reduction	5.00	0.00	0.00	100.00	
Preparation and recovery activities before and after exercise	5.00	0.00	0.00	100.00	
Symptoms and emergency treatment during exercise	5.00	0.00	0.00	100.00	
RICE principle	5.00	0.00	0.00	100.00	
Interactive Q&A and summary	5.00	0.00	0.00	100.00	
End of the program and thank you	5.00	0.00	0.00	100.00	

### 3.6 General evaluation

Throughout the evaluation of the overall effectiveness of the science popularization program, there was a clear trend of increasing consensus among the experts across all three rounds. Initially, there was considerable variability in the experts’ ratings of the program’s scientific nature and operationalization, as indicated by the higher standard deviations and coefficients of variation. By the second round, these measures began to decrease, reflecting a narrowing of expert opinions. In the final round, the experts reached a near-unanimous consensus on the clarity of the program’s theme, operationalization, and scientific nature, with the mean scores approaching or reaching 5.00 and the variability significantly reduced, demonstrating a strong endorsement of the program’s design and content (see Table 5).

**Table 5:** General evaluation of PARI public health education program after expert consultation of each round.

	The first round			The second round			The third round		
	Mean	*SD*	CV	Mean	*SD*	CV	Mean	*SD*	CV
Scientific nature of PARI public health education program	4.47	0.82	18.34	4.77	0.50	10.48	4.93	0.25	5.07
Operationalization of PARI public health education program	4.40	0.81	18.41	4.83	0.46	9.52	4.97	0.18	3.62
Clarity of the theme of PARI public health education program	4.83	0.46	9.52	4.93	0.25	5.07	5.00	0.00	0.00

## 4 Discussion

With the increasing prevalence of sports injuries among young people, particularly in the physically active college student population, it becomes necessary to implement effective measures to enhance their self-protection awareness (13, 14). In the rapidly evolving modern society, education plays multiple roles in cultural screening, transmission, transformation, critique, innovation, and reshaping (23). It is not merely a transmitter of knowledge, but also a shaper of ideas and values. Thus, preventive health education is particularly important. The PARI (Prevention, Awareness, Response, and Information) educational program aims to impart knowledge about sports injury prevention to college students. It not only enhances their understanding of sports safety but also helps them develop a positive attitude toward health and make appropriate responses when necessary.

Based on this, the study aims to design a PARI science popularization education plan for the college student population. We believe that through systematic educational intervention, it is possible to reduce the physical pain and economic burden caused by sports injuries. Additionally, it can promote students’ initiative in health management, help them establish scientific concepts of exercise, master the skills of preventing, identifying, and responding to sports injuries, and ultimately promote a healthy lifestyle among college students, laying a solid foundation for their long-term health and well-being.

### 4.1 Goals of programs

When designing a science popularization program aimed at enhancing college students’ knowledge and skills in preventing sports injuries, it is crucial to ensure the precision and relevance of the educational objectives. After three rounds of expert consultation using the Delphi method, our science popularization objectives were unanimously approved by the experts, with no objections. These objectives encompass the necessary professional knowledge and practical skills to ensure students not only understand the potential risks in sports but also are able to assess and respond when necessary.

The knowledge objectives focus on helping students understand potential risks in sports and recognize the role of regular health tests in maintaining good physical condition and preventing injuries. These objectives aim to ensure students can take the right actions before and after sports to reduce the risk of injury and recognize discomfort during activities, which is crucial for taking necessary preventive measures. Through this knowledge transfer, students will be better able to protect themselves and reduce unnecessary injuries caused by sports. In terms of skill objectives, we focus on teaching practical skills, such as the RICE principle in emergency situations – Rest, Ice, Compression, Elevation – which are crucial in handling sports injuries. Additionally, we emphasize the practice of health tests by students, and the importance of activities before and after exercise. These are key skills to help students self-manage and reduce the risk of injuries in daily sports. Thus, our science popularization program ensures that students not only theoretically understand the principles of preventing sports injuries but also apply the learned knowledge and skills in practice (24, 25).

### 4.2 Contents of programs

In content development, after three rounds of Delphi query, experts’ high recognition of certain items reflects a common understanding of their educational value and practicality. For instance, the monitoring of ‘heart rate,’ ‘blood pressure,’ and ‘cardiovascular diseases’ received unanimous emphasis, not only due to their concreteness and measurability but also their proven role in preventing cardiovascular-related diseases (26). These objective indicators are ideal for public health education, being easy to teach and convenient for students to grasp and apply. The retention of certain indicators in physical fitness tests, such as ‘cardiopulmonary endurance tests’ and ‘muscle strength and endurance tests,’ is because these tests help students understand their physical condition better, thus better guiding individual preventive PARI methods (27). These tests are seen as key tools in promoting self-health management and improving life quality, aligning with the current educational goal of enhancing individual health responsibility.

However, some items, such as ‘recognizing symptoms in physical activities’ and ‘monitoring kidney diseases,’ were removed due to their potential lack of direct relevance or practical application scenarios. For example, ‘recognizing symptoms in physical activities’ is too broad and difficult to operationalize, involving many subjective judgment factors, contrary to the clarity and empiricism sought in science popularization education. ‘Monitoring kidney diseases’ might be too specialized, challenging for non-professionals to understand and operate, limiting its practicality in popular education. Similarly, items like ‘dynamic stretching’ and ‘professional warm-up,’ suggested for removal in the second round, may be due to their limited general applicability and practicality for regular students, though beneficial for professional athletes (28). The purpose of science popularization education is to disseminate knowledge and improve the public’s overall health level, so content selection should lean toward those beneficial and easy to understand and implement for most people.

In conclusion, when constructing the PARI science popularization education program, content with a clear scientific basis, easy to popularize, and capable of enhancing students’ self-health management abilities should be chosen. At the same time, we need to consider the universality and practicality of the projects, avoiding those that may cause confusion or implementation difficulties. Thus, an effective PARI science popularization content is constructed.

### 4.3 Teaching methods of programs

In selecting teaching methods, we rigorously refined through the Delphi process. Initially considered methods included role-playing, interactive surveys, expert lectures, storytelling, field trips, and peer teaching. However, these were excluded in the second round as they were not quite aligned with the objectives of the program for undergraduates. Role-playing and storytelling, while interactive, might not fully cover the depth of the program’s content (29, 30). Surveys and lectures might render students’ passive participants, and field trips and peer teaching posed logistical challenges and lacked standardization (31, 32). Instead, the group unanimously agreed to adopt methods such as workshops, video analysis, and experiential learning, which encourage active student participation and are more conducive to undergraduate learning. These methods were unanimously affirmed in the third round, reflecting their suitability for the PARI program’s educational objectives.

Workshops provide a practical, collaborative environment where students can engage with materials in a real-world context, applying and reinforcing knowledge immediately. Video analysis and discussion allow participants to visually dissect and discuss various scenarios, deepening their understanding of the nuances in sports injuries and prevention (33). Experiential learning is vital as it transcends traditional didactic teaching, enabling students to consolidate knowledge through direct experience and reflection (34). Infographics and visual aids cater to different learning preferences, distilling complex information into digestible, visually appealing formats, thus enhancing memory and understanding (35). The inclusion of online courses and resources reflects the current state of modern education, providing flexible, convenient learning opportunities, aligning with undergraduates’ digital fluency (36). Lastly, reflection and journals serve as tools for self-assessment and continuous learning, encouraging students to articulate their understanding and issues encountered during the learning process (37).

In summary, the teaching methods chosen for the PARI project align with contemporary educational principles, prioritizing active learning, student engagement, and diverse modes of knowledge acquisition.

### 4.4 Time arrangement of programs

In terms of time allocation for science popularization activities, a meticulous evaluation using the Delphi method led to a carefully designed distribution of time for each part of the PARI program, which ultimately received unanimous approval in the third round. The time allocation for sections like ‘Activity Opening and Introduction,’ ‘Risk and Indicators of Cardiovascular Events in Sports,’ and ‘Physical Fitness Testing and Risk Reduction’ reflects a reasonable balance between comprehensive coverage and focused learning. Experts allocated ample time to key parts such as ‘Symptoms and Emergency Handling in Sports’ and ‘Risk and Indicators of Cardiovascular Events During Sports’ because it is essential for students to fully grasp and practice these lifesaving procedures. In contrast, less time was allocated to sections like ‘Participant Questions’ and ‘Key Points Summary,’ recognizing their auxiliary role in reinforcing the main content. This rational time arrangement also considers the cognitive load theory, ensuring students are not overwhelmed by information in a single session (38). By reasonably allocating time across different teaching activities, the program facilitates better mastery and understanding of knowledge among students. Additionally, the time allocated for ‘Interactive Q&A and Summary’ is sufficient for students to engage in active discussions, clarify doubts, and consolidate understanding, which is crucial for adult learners who benefit immensely from interactive and reflective learning stages.

## 5 Conclusion

The consensus reached through this Delphi study on the structure of public health education programs for undergraduates lays a crucial foundation for the development of informed and effective health promotion strategies within this demographic. It contributes significantly to the cultivation of health literacy and the establishment of proactive wellness practices among young adults. Additionally, the outcomes of this research can aid educational institutions in tailoring their health education curricula to better suit the needs and dynamics of undergraduate populations.

This study designed a highly comprehensive PARI science popularization education program for Chinese undergraduates, thereby gradually enhancing the health literacy and self-protection capabilities of Chinese youth against physical injuries, as well as safeguarding their physical health. Moreover, this research lays a foundation for similar future science popularization programs and serves as a model for similar science popularization programs abroad.

## Data availability statement

The raw data supporting the conclusions of this article will be made available by the authors, without undue reservation.

## Ethics statement

The studies involving humans were approved by Ethics Committee on Third Xiangya Hospital of Central South University. The studies were conducted in accordance with the local legislation and institutional requirements. The participants provided their written informed consent to participate in this study.

## Author contributions

YK: Formal analysis, Funding acquisition, Validation, Visualization, Writing – original draft, Writing – review & editing. HX: Writing – original draft. CL: Data curation, Formal analysis, Methodology, Writing – original draft. YY: Investigation, Methodology, Writing – original draft. XZ: Investigation, Writing – original draft. YZ: Project administration, Supervision, Writing – review & editing.
