# Supplementary material for: Construction of PARI public health education programs for Chinese undergraduates: a Delphi study
Source: Front Public Health. 2024 Jun 17;12:1390011. doi: 10.3389/fpubh.2024.1390011 (PMC11215213; doi:10.3389/fpubh.2024.1390011)
Supplement: Supplementary file 1 [file Table_1.DOCX]

Table S1 Goals of PARI public health education program after expert consultation (First-round)

| Items of First-Level | Items of Second-Level | Mean | SD | CV | Notes |
| --- | --- | --- | --- | --- | --- |
| Knowledge goals | Recognizing risks in sports | 4.97 | 0.18 | 3.62 |  |
|  | Understanding the importance of fitness testing | 4.87 | 0.43 | 8.83 |  |
|  | Knowing the correct way to move before and after exercise | 5.00 | 0.00 | 0.00 |  |
|  | Recognize discomfort during exercise | 5.00 | 0.00 | 0.00 |  |
|  | Learn the RICE principle of emergency treatment of sports injuries | 4.97 | 0.18 | 3.62 |  |
| Skill goals | Conducting fitness tests | 4.87 | 0.35 | 7.19 |  |
|  | Practice of pre- and post-exercise activities | 5.00 | 0.00 | 0.00 |  |
|  | Self-assessment and treatment | 5.00 | 0.00 | 0.00 |  |
|  | Emergency management skills | 5.00 | 0.00 | 0.00 |  |
|  | Health awareness and self-management | 5.00 | 0.00 | 0.00 |  |

Table S2 Goals of PARI public health education program after expert consultation (Second-round)

| Items of First-Level | Items of Second-Level | Mean | SD | CV | AR | Notes |
| --- | --- | --- | --- | --- | --- | --- |
| Professional knowledge goal | Recognizing risks in sports | 5.00 | 0.00 | 0.00 | 100.00 |  |
|  | Understanding the importance of fitness testing | 4.97 | 0.18 | 3.62 | 100.00 |  |
|  | Knowing the correct way to move before and after exercise | 5.00 | 0.00 | 0.00 | 100.00 |  |
|  | Recognize discomfort during exercise | 5.00 | 0.00 | 0.00 | 100.00 |  |
|  | Learn the RICE principle of emergency treatment of sports injuries | 5.00 | 0.00 | 0.00 | 100.00 |  |
| Skill goals | Conducting fitness tests | 4.97 | 0.18 | 3.62 | 100.00 |  |
|  | Practice of pre- and post-exercise activities | 5.00 | 0.00 | 0.00 | 100.00 |  |
|  | Self-assessment and treatment | 5.00 | 0.00 | 0.00 | 100.00 |  |
|  | Emergency management skills | 5.00 | 0.00 | 0.00 | 100.00 |  |
|  | Health awareness and self-management | 5.00 | 0.00 | 0.00 | 100.00 |  |

Table S3 Goals of PARI public health education program after expert consultation (Third-round)

| Items of First-Level | Items of Second-Level | Mean | SD | CV | AR | Notes |
| --- | --- | --- | --- | --- | --- | --- |
| Professional knowledge goal | Recognizing risks in sports | 5.00 | 0.00 | 0.00 | 100.00 |  |
|  | Understanding the importance of fitness testing | 5.00 | 0.00 | 0.00 | 100.00 |  |
|  | Knowing the correct way to move before and after exercise | 5.00 | 0.00 | 0.00 | 100.00 |  |
|  | Recognize discomfort during exercise | 5.00 | 0.00 | 0.00 | 100.00 |  |
|  | Learn the RICE principle of emergency treatment of sports injuries | 5.00 | 0.00 | 0.00 | 100.00 |  |
| Skill goals | Conducting fitness tests | 5.00 | 0.00 | 0.00 | 100.00 |  |
|  | Practice of pre- and post-exercise activities | 5.00 | 0.00 | 0.00 | 100.00 |  |
|  | Self-assessment and treatment | 5.00 | 0.00 | 0.00 | 100.00 |  |
|  | Emergency management skills | 5.00 | 0.00 | 0.00 | 100.00 |  |
|  | Health awareness and self-management | 5.00 | 0.00 | 0.00 | 100.00 |  |

Table S4 Contents of PARI public health education program after expert consultation (First-round)

| First-Level Item | Second-Level Item | Third-Level Items | Mean | SD | CV | Notes |
| --- | --- | --- | --- | --- | --- | --- |
| Risk Indicators in Sports |  |  | 4.73 | 0.58 | 12.26 |  |
|  | Physical Activity Levels |  | 4.37 | 0.67 | 15.33 |  |
|  |  | Definitions and Importance | 4.50 | 0.57 | 12.67 | Modify(R5) |
|  |  | How to Measure | 4.57 | 0.50 | 10.94 |  |
|  |  | Recommended Criteria | 4.27 | 0.69 | 16.16 |  |
|  |  | Strategies for Increasing Physical Activity Levels | 4.53 | 0.51 | 11.26 |  |
|  | Routine Medical Tests |  | 4.20 | 0.76 | 18.10 |  |
|  |  | Heart rate | 4.93 | 0.25 | 5.07 |  |
|  |  | Blood Pressure | 4.90 | 0.31 | 6.33 |  |
|  |  | Cardiovascular Disease | 4.97 | 0.18 | 3.62 |  |
|  | Recognizing Signs and Symptoms |  | 2.23 | 0.94 | 42.15 | Delete (R1, R2) |
|  |  | How to Measure | 4.63 | 0.56 | 12.10 | Delete (R1, R2, R+) |
|  |  | Kidney Disease | 2.53 | 0.78 | 30.83 | Delete (R1, R2, R+) |
|  |  | How to respond | 4.53 | 0.63 | 13.91 | Delete(R+) |
| Fitness Tests |  |  | 4.47 | 0.82 | 18.34 |  |
|  | Importance of fitness testing |  | 4.43 | 0.77 | 17.38 |  |
|  |  | Risk Assessment | 4.70 | 0.60 | 12.77 | Modify(R5) |
|  |  | Personalized Exercise Plans | 4.97 | 0.18 | 3.62 |  |
|  |  | Monitoring Progress | 4.07 | 1.20 | 29.48 | Delete(R2,R4) |
|  | Types of Fitness Tests |  | 4.53 | 0.51 | 11.26 |  |
|  |  | Cardiorespiratory Endurance Testing | 4.93 | 0.25 | 5.07 |  |
|  |  | Muscular strength and endurance testing | 4.93 | 0.25 | 5.07 |  |
|  |  | Flexibility Testing | 4.90 | 0.31 | 6.33 |  |
|  |  | Body Composition Testing | 5.00 | 0.00 | 0.00 |  |
|  | How to Prepare for a Fitness Test |  | 4.63 | 0.49 | 10.58 |  |
|  |  | Physical Preparation | 4.97 | 0.18 | 3.62 |  |
|  |  | Diet and hydration | 4.97 | 0.18 | 3.62 |  |
|  |  | Mental Preparation | 4.90 | 0.31 | 6.33 |  |
|  | Interpreting Fitness Test Results |  | 4.73 | 0.69 | 14.59 |  |
|  |  | Understanding Results | 4.90 | 0.31 | 6.33 |  |
|  |  | Setting Goals | 4.90 | 0.31 | 6.33 |  |
|  |  | Adjusting Exercise Programs | 4.93 | 0.25 | 5.07 |  |
|  | The Importance of Regular Fitness Testing |  | 4.13 | 0.73 | 17.68 |  |
|  |  | Ongoing Monitoring | 4.73 | 0.58 | 12.26 | Modify(R6) |
|  |  | Adjusting Strategies | 4.70 | 0.65 | 13.83 | Modify(R6) |
| Activities before and after exercise |  |  | 4.70 | 0.47 | 10.00 |  |
|  | The Importance of Warming Up |  | 4.20 | 0.71 | 16.91 |  |
|  |  | Preventing Injury | 5.00 | 0.00 | 0.00 |  |
|  |  | Improving Performance | 4.93 | 0.31 | 6.29 |  |
|  |  | Mental Preparation | 4.50 | 0.78 | 17.33 |  |
|  | Types of Warm-Up Activities |  | 4.20 | 0.76 | 18.10 |  |
|  |  | General Warm-up | 4.97 | 0.18 | 3.62 |  |
|  |  | Specialized Warm-Up | 4.97 | 0.18 | 3.62 |  |
|  |  | Dynamic Stretching | 4.97 | 0.18 | 3.62 |  |
|  | The Importance of Post-Exercise Finishing Activities |  | 4.20 | 0.81 | 19.29 |  |
|  |  | Promoting Recovery | 5.00 | 0.00 | 0.00 |  |
|  |  | Preventing Stiffness | 5.00 | 0.00 | 0.00 |  |
|  |  | Smooth transition of heart rate and breathing | 5.00 | 0.00 | 0.00 |  |
|  | Types of Post-Exercise Stretching Exercises |  | 4.20 | 0.81 | 19.29 |  |
|  |  | Static Stretching | 4.97 | 0.18 | 3.62 |  |
|  |  | Relaxes muscles | 4.93 | 0.31 | 6.29 |  |
|  |  | Improves flexibility | 4.97 | 0.18 | 3.62 |  |
|  | Safety Precautions |  | 4.53 | 0.73 | 16.11 |  |
|  |  | Avoid overstretching | 5.00 | 0.00 | 0.00 |  |
|  |  | Maintain breathing | 5.00 | 0.00 | 0.00 |  |
|  |  | Duration and Frequency | 5.00 | 0.00 | 0.00 |  |
| Handling of discomfort during exercise |  |  | 4.57 | 0.68 | 14.88 |  |
|  | Knowing and Recognizing Discomfort |  | 4.63 | 0.56 | 12.10 |  |
|  |  | Chest pain | 5.00 | 0.00 | 0.00 |  |
|  |  | Palpitations | 5.00 | 0.00 | 0.00 |  |
|  |  | Muscle and joint pain | 5.00 | 0.00 | 0.00 |  |
|  | Immediate Steps to Take |  | 4.63 | 0.56 | 12.10 |  |
|  |  | Stop Exercising | 5.00 | 0.00 | 0.00 |  |
|  |  | Seek help | 5.00 | 0.00 | 0.00 |  |
|  |  | Conduct a self-assessment | 5.00 | 0.00 | 0.00 |  |
|  | When to Seek Medical Help |  | 4.20 | 0.76 | 18.10 |  |
|  |  | Emergencies | 5.00 | 0.00 | 0.00 |  |
|  |  | Make an appointment to see a doctor | 1.07 | 0.25 | 23.36 | Delete (R1, R2, R4) |
|  | Preventive Measures |  | 4.63 | 0.56 | 12.10 |  |
|  |  | Professional Evaluation | 4.93 | 0.25 | 5.07 |  |
|  |  | Warm up properly | 1.07 | 0.25 | 23.36 | Delete (R1, R2, R4) |
|  |  | Gradual increase in training | 4.77 | 0.50 | 10.48 |  |
|  |  | Regular medical checkups | 4.90 | 0.40 | 8.16 |  |
|  | Recovery and Rehabilitation |  | 4.63 | 0.56 | 12.10 |  |
|  |  | Rest | 4.97 | 0.18 | 3.62 |  |
|  |  | Gradual Recovery | 5.00 | 0.00 | 0.00 |  |
|  |  | Tracking Symptoms | 5.00 | 0.00 | 0.00 |  |
| RICE principle content and application |  |  | 4.93 | 0.25 | 5.07 |  |
|  | Introduction to the RICE principle |  | 4.97 | 0.18 | 3.62 |  |
|  |  | What is the RICE Principle | 4.97 | 0.18 | 3.62 |  |
|  |  | Why use the RICE principle | 4.93 | 0.25 | 5.07 |  |
|  | Rest |  | 4.90 | 0.31 | 6.33 |  |
|  |  | Stopping Activity | 5.00 | 0.00 | 0.00 |  |
|  |  | Protecting the Injured Area | 5.00 | 0.00 | 0.00 |  |
|  | Ice |  | 4.90 | 0.31 | 6.33 |  |
|  |  | The Right Way to Apply Ice | 5.00 | 0.00 | 0.00 |  |
|  |  | Benefits of Icing | 5.00 | 0.00 | 0.00 |  |
|  | Compression |  | 4.90 | 0.31 | 6.33 |  |
|  |  | Using an Elastic Bandage | 5.00 | 0.00 | 0.00 |  |
|  |  | Precautions | 5.00 | 0.00 | 0.00 |  |
|  | Elevation |  | 4.90 | 0.31 | 6.33 |  |
|  |  | Elevate the injured area | 5.00 | 0.00 | 0.00 |  |
|  |  | Appropriate height | 4.93 | 0.25 | 5.07 |  |
|  | Seek medical attention and follow-up |  | 4.13 | 0.73 | 17.68 |  |
|  |  | When to seek professional help | 4.80 | 0.41 | 8.54 |  |
|  |  | Recovery and Rehabilitation | 3.63 | 0.56 | 15.43 | Delete (R1, R4) |

Table S5 Contents of PARI public health education program after expert consultation (Second-round)

| First-Level Item | Second-Level Item | Third-Level Items | Mean | SD | CV | AR | Notes |
| --- | --- | --- | --- | --- | --- | --- | --- |
| Risk Indicators in Sports |  |  | 4.90 | 0.31 | 6.33 | 93.33 |  |
|  | Physical Activity Levels |  | 4.50 | 0.63 | 14.00 | 90.00 |  |
|  |  | Definitions and Importance | 4.57 | 0.50 | 10.94 | 93.33 |  |
|  |  | How to Measure | 4.67 | 0.48 | 10.28 | 90.00 |  |
|  |  | Recommended Criteria | 4.53 | 0.57 | 12.58 | 96.67 |  |
|  |  | Strategies for Increasing Physical Activity Levels | 4.67 | 0.48 | 10.28 | 100.00 |  |
|  | Routine Medical Tests |  | 4.53 | 0.63 | 13.91 | 90.00 |  |
|  |  | Heart rate | 4.97 | 0.18 | 3.62 | 100.00 |  |
|  |  | Blood Pressure | 5.00 | 0.00 | 0.00 | 100.00 |  |
|  |  | Cardiovascular Disease | 4.97 | 0.18 | 3.62 | 100.00 |  |
| Fitness Tests |  |  | 4.70 | 0.53 | 11.28 | 83.33 |  |
|  | Importance of fitness testing |  | 4.63 | 0.72 | 15.55 | 90.00 |  |
|  |  | Risk Assessment | 4.90 | 0.31 | 6.33 | 96.67 |  |
|  |  | Personalized Exercise Plans | 5.00 | 0.00 | 0.00 | 100.00 |  |
|  | Types of Fitness Tests |  | 4.63 | 0.49 | 10.58 | 100.00 |  |
|  |  | Cardiorespiratory Endurance Testing | 4.93 | 0.25 | 5.07 | 100.00 |  |
|  |  | Muscular strength and endurance testing | 4.93 | 0.25 | 5.07 | 100.00 |  |
|  |  | Flexibility Testing | 4.93 | 0.25 | 5.07 | 100.00 |  |
|  |  | Body Composition Testing | 5.00 | 0.00 | 0.00 | 100.00 |  |
|  | How to Prepare for a Fitness Test |  | 4.90 | 0.31 | 6.33 | 100.00 |  |
|  |  | Physical Preparation | 4.97 | 0.18 | 3.62 | 100.00 |  |
|  |  | Diet and hydration | 5.00 | 0.00 | 0.00 | 100.00 |  |
|  |  | Mental Preparation | 4.97 | 0.18 | 3.62 | 100.00 |  |
|  | Interpreting Fitness Test Results |  | 4.93 | 0.25 | 5.07 | 100.00 |  |
|  |  | Understanding Results | 4.93 | 0.25 | 5.07 | 100.00 |  |
|  |  | Setting Goals | 4.97 | 0.18 | 3.62 | 100.00 |  |
|  |  | Adjusting Exercise Programs | 4.97 | 0.18 | 3.62 | 100.00 |  |
|  | The Importance of Regular Fitness Testing |  | 4.90 | 0.31 | 6.33 | 100.00 |  |
|  |  | Ongoing Monitoring | 4.90 | 0.31 | 6.33 | 100.00 |  |
|  |  | Adjusting Strategies | 4.90 | 0.31 | 6.33 | 96.67 |  |
| Activities before and after exercise |  |  | 4.83 | 0.46 | 9.52 | 90.00 |  |
|  | The Importance of Warming Up |  | 4.00 | 0.57 | 12.67 | 96.67 |  |
|  |  | Preventing Injury | 4.97 | 0.18 | 3.62 | 100.00 |  |
|  |  | Improving Performance | 4.93 | 0.25 | 5.07 | 100.00 |  |
|  |  | Mental Preparation | 4.77 | 0.43 | 9.01 | 96.33 |  |
|  | Types of Warm-Up Activities |  | 4.53 | 0.68 | 14.35 | 96.33 |  |
|  |  | General Warm-up | 5.00 | 0.00 | 0.00 | 100.00 |  |
|  |  | Specialized Warm-Up | 4.17 | 1.29 | 30.91 | 73.33 | Delete (R2, R3, R4) |
|  |  | Dynamic Stretching | 4.17 | 1.29 | 30.91 | 73.33 | Delete (R2, R3, R4) |
|  | The Importance of Post-Exercise Finishing Activities |  | 4.53 | 0.68 | 14.35 | 96.33 |  |
|  |  | Promoting Recovery | 4.93 | 0.25 | 5.07 | 100.00 |  |
|  |  | Preventing Stiffness | 4.97 | 0.18 | 3.62 | 100.00 |  |
|  |  | Smooth transition of heart rate and breathing | 4.97 | 0.18 | 3.62 | 100.00 |  |
|  | Types of Post-Exercise Stretching Exercises |  | 4.63 | 0.67 | 14.47 | 96.67 |  |
|  |  | Static Stretching | 4.93 | 0.25 | 5.07 | 96.67 |  |
|  |  | Relaxes muscles | 4.90 | 0.31 | 6.33 | 93.33 |  |
|  |  | Improves flexibility | 4.93 | 0.25 | 5.07 | 96.67 |  |
|  | Safety Precautions |  | 4.63 | 0,67 | 14.47 | 96.33 |  |
|  |  | Avoid overstretching | 4.97 | 0.18 | 3.62 | 100.00 |  |
|  |  | Maintain breathing | 5.00 | 0.00 | 0.00 | 100.00 |  |
|  |  | Duration and Frequency | 5.00 | 0.00 | 0.00 | 100.00 |  |
| Handling of discomfort during exercise |  |  | 4.73 | 0.52 | 10.99 | 86.67 |  |
|  | Knowing and Recognizing Discomfort |  | 4.73 | 0.52 | 10.99 | 86.67 |  |
|  |  | Chest pain | 5.00 | 0.00 | 0.00 | 100.00 |  |
|  |  | Palpitations | 5.00 | 0.00 | 0.00 | 100.00 |  |
|  |  | Muscle and joint pain | 5.00 | 0.00 | 0.00 | 100.00 |  |
|  | Immediate Steps to Take |  | 5.00 | 0.00 | 0.00 | 100.00 |  |
|  |  | Stop Exercising | 4.97 | 0.18 | 3.62 | 100.00 |  |
|  |  | Seek help | 4.93 | 0.25 | 5.07 | 100.00 |  |
|  |  | Conduct a self-assessment | 4.93 | 0.25 | 5.07 | 100.00 |  |
|  | When to Seek Medical Help |  | 4.73 | 0.52 | 10.99 | 86.67 |  |
|  |  | Emergencies | 4.93 | 0.25 | 5.07 | 96.67 | Modify(R6) |
|  | Preventive Measures |  | 5.00 | 0.00 | 0.00 | 100.00 |  |
|  |  | Professional Evaluation | 4.33 | 1.09 | 25.17 | 76.67 | Delete (R2, R3, R4) |
|  |  | Gradual increase in training | 4.83 | 0.38 | 7.87 | 90.00 |  |
|  |  | Regular medical checkups | 5.00 | 0.00 | 0.00 | 100.00 |  |
|  | Recovery and Rehabilitation |  | 5.00 | 0.00 | 0.00 | 100.00 |  |
|  |  | Rest | 5.00 | 0.00 | 0.00 | 100.00 |  |
|  |  | Gradual Recovery | 5.00 | 0.00 | 0.00 | 100.00 |  |
|  |  | Tracking Symptoms | 5.00 | 0.00 | 0.00 | 100.00 |  |
| RICE principle content and application |  |  | 5.00 | 0.00 | 0.00 | 100.00 |  |
|  | Introduction to the RICE principle |  | 5.00 | 0.00 | 0.00 | 100.00 |  |
|  |  | What is the RICE Principle | 5.00 | 0.00 | 0.00 | 100.00 |  |
|  |  | Why use the RICE principle | 5.00 | 0.00 | 0.00 | 100.00 |  |
|  | Rest |  | 5.00 | 0.00 | 0.00 | 100.00 |  |
|  |  | Stopping Activity | 4.97 | 0.18 | 3.62 | 100.00 |  |
|  |  | Protecting the Injured Area | 4.93 | 0.25 | 5.07 | 100.00 |  |
|  | Ice |  | 5.00 | 0.00 | 0.00 | 100.00 |  |
|  |  | The Right Way to Apply Ice | 4.93 | 0.25 | 5.07 | 100.00 |  |
|  |  | Benefits of Icing | 4.90 | 0.31 | 6.33 | 100.00 |  |
|  | Compression |  | 5.00 | 0.00 | 0.00 | 100.00 |  |
|  |  | Using an Elastic Bandage | 4.93 | 0.25 | 5.07 | 100.00 |  |
|  |  | Precautions | 5.00 | 0.00 | 0.00 | 100.00 |  |
|  | Elevation |  | 5.00 | 0.00 | 0.00 | 100.00 |  |
|  |  | Elevate the injured area | 5.00 | 0.00 | 0.00 | 100.00 |  |
|  |  | Appropriate height | 4.97 | 0.18 | 3.62 | 100.00 |  |
|  | Seek medical attention and follow-up |  | 5.00 | 0.00 | 0.00 | 100.00 |  |
|  |  | When to seek professional help | 4.87 | 0.35 | 7.19 | 100.00 |  |

Table S6 Contents of PARI public health education program after expert consultation (Third-round)

| First-Level Item | Second-Level Item | Third-Level Items | Mean | SD | CV | AR | Notes |
| --- | --- | --- | --- | --- | --- | --- | --- |
| Risk Indicators in Sports |  |  | 5.00 | 0.00 | 0.00 | 100.00 |  |
|  | Physical Activity Levels |  | 4.90 | 0.31 | 6.33 | 100.00 |  |
|  |  | Definitions and Importance | 4.90 | 0.31 | 6.33 | 100.00 |  |
|  |  | How to Measure | 5.00 | 0.00 | 0.00 | 100.00 |  |
|  |  | Recommended Criteria | 5.00 | 0.00 | 0.00 | 100.00 |  |
|  |  | Strategies for Increasing Physical Activity Levels | 5.00 | 0.00 | 0.00 | 100.00 |  |
|  | Routine Medical Tests |  | 4.90 | 0.31 | 6.33 | 100.00 |  |
|  |  | Heart rate | 5.00 | 0.00 | 0.00 | 100.00 |  |
|  |  | Blood Pressure | 5.00 | 0.00 | 0.00 | 100.00 |  |
|  |  | Cardiovascular Disease | 5.00 | 0.00 | 0.00 | 100.00 |  |
| Fitness Tests |  |  | 4.93 | 0.25 | 5.07 | 96.67 |  |
|  | Importance of fitness testing |  | 4.90 | 0.31 | 6.33 | 100.00 |  |
|  |  | Risk Assessment | 4.93 | 0.25 | 5.07 | 96.67 |  |
|  |  | Personalized Exercise Plans | 5.00 | 0.00 | 0.00 | 100.00 |  |
|  | Types of Fitness Tests |  | 4.90 | 0.31 | 6.33 | 100.00 |  |
|  |  | Cardiorespiratory Endurance Testing | 5.00 | 0.00 | 0.00 | 100.00 |  |
|  |  | Muscular strength and endurance testing | 5.00 | 0.00 | 0.00 | 100.00 |  |
|  |  | Flexibility Testing | 5.00 | 0.00 | 0.00 | 100.00 |  |
|  |  | Body Composition Testing | 5.00 | 0.00 | 0.00 | 100.00 |  |
|  | How to Prepare for a Fitness Test |  | 5.00 | 0.00 | 0.00 | 100.00 |  |
|  |  | Physical Preparation | 5.00 | 0.00 | 0.00 | 100.00 |  |
|  |  | Diet and hydration | 5.00 | 0.00 | 0.00 | 100.00 |  |
|  |  | Mental Preparation | 5.00 | 0.00 | 0.00 | 100.00 |  |
|  | Interpreting Fitness Test Results |  | 5.00 | 0.00 | 0.00 | 100.00 |  |
|  |  | Understanding Results | 5.00 | 0.00 | 0.00 | 100.00 |  |
|  |  | Setting Goals | 5.00 | 0.00 | 0.00 | 100.00 |  |
|  |  | Adjusting Exercise Programs | 5.00 | 0.00 | 0.00 | 100.00 |  |
|  | The Importance of Regular Fitness Testing |  | 4.90 | 0.31 | 6.33 | 100.00 |  |
|  |  | Ongoing Monitoring | 5.00 | 0.00 | 0.00 | 100.00 |  |
|  |  | Adjusting Strategies | 5.00 | 0.00 | 0.00 | 100.00 |  |
| Activities before and after exercise |  |  | 5.00 | 0.00 | 0.00 | 96.67 |  |
|  | The Importance of Warming Up |  | 4.93 | 0.25 | 5.07 | 100.00 |  |
|  |  | Preventing Injury | 4.97 | 0.18 | 3.62 | 100.00 |  |
|  |  | Improving Performance | 4.97 | 0.18 | 3.62 | 100.00 |  |
|  |  | Mental Preparation | 4.97 | 0.18 | 3.62 | 100.00 |  |
|  | Types of Warm-Up Activities |  | 4.87 | 0.35 | 7.19 | 100.00 |  |
|  |  | General Warm-up | 5.00 | 0.00 | 0.00 | 100.00 |  |
|  | The Importance of Post-Exercise Finishing Activities |  | 4.90 | 0.31 | 6.33 | 100.00 |  |
|  |  | Promoting Recovery | 5.00 | 0.00 | 0.00 | 100.00 |  |
|  |  | Preventing Stiffness | 5.00 | 0.00 | 0.00 | 100.00 |  |
|  |  | Smooth transition of heart rate and breathing | 5.00 | 0.00 | 0.00 | 100.00 |  |
|  | Types of Post-Exercise Stretching Exercises |  | 4.90 | 0.31 | 6.33 | 100.00 |  |
|  |  | Static Stretching | 5.00 | 0.00 | 0.00 | 100.00 |  |
|  |  | Relaxes muscles | 5.00 | 0.00 | 0.00 | 100.00 |  |
|  |  | Improves flexibility | 5.00 | 0.00 | 0.00 | 100.00 |  |
|  | Safety Precautions |  | 4.97 | 0.18 | 3.62 | 100.00 |  |
|  |  | Avoid overstretching | 5.00 | 0.00 | 0.00 | 100.00 |  |
|  |  | Maintain breathing | 5.00 | 0.00 | 0.00 | 100.00 |  |
|  |  | Duration and Frequency | 5.00 | 0.00 | 0.00 | 100.00 |  |
| Handling of discomfort during exercise |  |  | 4.93 | 0.25 | 5.07 | 96.67 |  |
|  | Knowing and Recognizing Discomfort |  | 5.00 | 0.00 | 0.00 | 100.00 |  |
|  |  | Chest pain | 5.00 | 0.00 | 0.00 | 100.00 |  |
|  |  | Palpitations | 5.00 | 0.00 | 0.00 | 100.00 |  |
|  |  | Muscle and joint pain | 5.00 | 0.00 | 0.00 | 100.00 |  |
|  | Immediate Steps to Take |  | 5.00 | 0.00 | 0.00 | 100.00 |  |
|  |  | Stop Exercising | 5.00 | 0.00 | 0.00 | 100.00 |  |
|  |  | Seek help | 5.00 | 0.00 | 0.00 | 100.00 |  |
|  |  | Conduct a self-assessment | 5.00 | 0.00 | 0.00 | 100.00 |  |
|  | When to Seek Medical Help |  | 5.00 | 0.00 | 0.00 | 100.00 |  |
|  |  | Emergencies | 5.00 | 0.00 | 0.00 | 100.00 |  |
|  | Preventive Measures |  | 5.00 | 0.00 | 0.00 | 100.00 |  |
|  |  | Gradual increase in training | 4.97 | 0.18 | 3.62 | 100.00 |  |
|  |  | Regular medical checkups | 5.00 | 0.00 | 0.00 | 100.00 |  |
|  | Recovery and Rehabilitation |  | 5.00 | 0.00 | 0.00 | 100.00 |  |
|  |  | Rest | 5.00 | 0.00 | 0.00 | 100.00 |  |
|  |  | Gradual Recovery | 5.00 | 0.00 | 0.00 | 100.00 |  |
|  |  | Tracking Symptoms | 5.00 | 0.00 | 0.00 | 100.00 |  |
| RICE principle content and application |  |  | 5.00 | 0.00 | 0.00 | 100.00 |  |
|  | Introduction to the RICE principle |  | 4.93 | 0.25 | 5.07 | 100.00 |  |
|  |  | What is the RICE Principle | 5.00 | 0.00 | 0.00 | 100.00 |  |
|  |  | Why use the RICE principle | 5.00 | 0.00 | 0.00 | 100.00 |  |
|  | Rest |  | 5.00 | 0.00 | 0.00 | 100.00 |  |
|  |  | Stopping Activity | 5.00 | 0.00 | 0.00 | 100.00 |  |
|  |  | Protecting the Injured Area | 5.00 | 0.00 | 0.00 | 100.00 |  |
|  | Ice |  | 5.00 | 0.00 | 0.00 | 100.00 |  |
|  |  | The Right Way to Apply Ice | 5.00 | 0.00 | 0.00 | 100.00 |  |
|  |  | Benefits of Icing | 5.00 | 0.00 | 0.00 | 100.00 |  |
|  | Compression |  | 5.00 | 0.00 | 0.00 | 100.00 |  |
|  |  | Using an Elastic Bandage | 5.00 | 0.00 | 0.00 | 100.00 |  |
|  |  | Precautions | 5.00 | 0.00 | 0.00 | 100.00 |  |
|  | Elevation |  | 5.00 | 0.00 | 0.00 | 100.00 |  |
|  |  | Elevate the injured area | 5.00 | 0.00 | 0.00 | 100.00 |  |
|  |  | Appropriate height | 5.00 | 0.00 | 0.00 | 100.00 |  |

Table S7 Teaching methods of PARI public health education program after expert consultation (Second-round)

| Items | Mean | SD | CV | AR | Notes |
| --- | --- | --- | --- | --- | --- |
| Role Play | 4.33 | 0.92 | 21.25 | 90.00 | Delete (R2, R4) |
| Interactive Questionnaire | 4.93 | 0.25 | 5.07 | 73.33 | Delete (R3, R4) |
| Sports Injury Prevention Workshop | 4.63 | 0.67 | 14.47 | 96.67 |  |
| Video Analysis and Discussion | 4.87 | 0.35 | 7.19 | 96.33 |  |
| Experiential Learning | 5.00 | 0.00 | 0.00 | 100.00 |  |
| Expert Lecture & Interaction | 2.13 | 1.07 | 50.23 | 33.33 | Delete (R1, R2, R3, R4) |
| Storytelling | 3.33 | 1.42 | 42.64 | 63.33 | Delete (R1, R2, R3) |
| Infographics and Visual Aids | 4.87 | 0.35 | 7.19 | 83.33 |  |
| Field trips or visits | 2.50 | 0.68 | 27.20 | 16.67 | Delete (R1, R2, R3, R4) |
| Online courses and resources | 4.97 | 0.18 | 3.62 | 86.67 |  |
| Reflections and journal entries | 4.50 | 0.51 | 11.33 | 100.00 |  |
| Peer teaching and sharing | 3.80 | 0.89 | 23.42 | 70.00 | Delete (R1, R2, R3, R4) |

Table S8 Teaching methods of PARI public health education program after expert consultation (Third-round)

| Items | Mean | SD | CV | AR | Notes |
| --- | --- | --- | --- | --- | --- |
| Sports Injury Prevention Workshop | 4.90 | 0.31 | 6.33 | 100.00 |  |
| Video Analysis and Discussion | 4.93 | 0.25 | 5.07 | 100.00 |  |
| Experiential Learning | 5.00 | 0.00 | 0.00 | 100.00 |  |
| Infographics and Visual Aids | 4.90 | 0.31 | 6.33 | 93.33 |  |
| Online courses and resources | 5.00 | 0.00 | 0.00 | 100.00 |  |
| Reflections and journal entries | 4.87 | 0.35 | 7.19 | 100.00 |  |
